# Polyparasite Helminth Infections and Their Association to Anaemia and Undernutrition in Northern Rwanda

**DOI:** 10.1371/journal.pntd.0000517

**Published:** 2009-09-15

**Authors:** Denise Mupfasoni, Blaise Karibushi, Artemis Koukounari, Eugene Ruberanziza, Teddy Kaberuka, Michael H. Kramer, Odette Mukabayire, Michee Kabera, Vianney Nizeyimana, Marie-Alice Deville, Josh Ruxin, Joanne P. Webster, Alan Fenwick

**Affiliations:** 1 Access Project, Kigali, Rwanda; 2 Schistosomiasis Control Initiative, Department of Infectious Disease Epidemiology, Imperial College London, London, United Kingdom; 3 TRAC Plus - Center for Treatment and Research on AIDS, Malaria, Tuberculosis and Other Epidemics, Kigali, Rwanda; 4 National Reference Laboratory, Kigali, Rwanda; 5 The Earth Institute, Columbia University, New York, New York, United States of America; Case Western Reserve University School of Medicine, United States of America

## Abstract

**Background:**

Intestinal schistosomiasis and soil-transmitted helminth (STH) infections constitute major public health problems in many parts of sub-Saharan Africa. In this study we examined the functional significance of such polyparasite infections in anemia and undernutrition in Rwandan individuals.

**Methods:**

Three polyparasite infection profiles were defined, in addition to a reference profile that consisted of either no infections or low-intensity infection with only one of the focal parasite species. Logistic regression models were applied to data of 1,605 individuals from 6 schools in 2 districts of the Northern Province before chemotherapeutic treatment in order to correctly identify individuals who were at higher odds of being anaemic and/or undernourished.

**Findings:**

Stunted relative to nonstunted, and males compared to females, were found to be at higher odds of being anaemic independently of polyparasite infection profile. The odds of being wasted were 2-fold greater for children with concurrent infection of at least 2 parasites at M+ intensity compared to those children with the reference profile. Males compared to females and anaemic compared to nonanaemic children were significantly more likely to be stunted. None of the three polyparasite infection profiles were found to have significant effects on stunting.

**Conclusion:**

The present data suggest that the levels of polyparasitism, and infection intensities in the Rwandan individuals examined here may be lower as compared to other recent similar epidemiological studies in different regions across sub-Saharan Africa. Neither the odds of anaemia nor the odds of stunting were found to be significantly different in the three-polyparasite infection profiles. However, the odds of wasting were higher in those children with at least two parasites at M+ intensity compared to those children with the reference profile. Nevertheless, despite the low morbidity levels indicated in the population under study here, we recommend sustainable efforts for the deworming of affected populations to be continued in order to support the economic development of the country.

## Author Summary

The helminth infections—schistosomiasis, hookworm, ascariasis and trichuriasis—are the main neglected tropical diseases (NTDs) to thrive in sub-Saharan Africa. Here we assess the distribution and the intensities of such polyparasite infections in two districts of the Northern Province in Rwanda and determine whether these are associated with anaemia, lowered haemoglobin levels and recent and/or chronic undernutrition. Rwanda is a small landlocked country in Central Africa where no research or control efforts on NTDs has been conducted since before the genocide in 1994. The current study aimed to elucidate, for the first time post-genocide, the burden of NTDs on the health of the Rwandan people and potential associated morbidity. Despite the fact that we observed low morbidity levels and intensities of polyparasite helminth infections, we recommend sustainable efforts for the deworming of the Rwandan people to be continued in order to offer a worm-free physical and cognitive development to the children of Rwanda and hence support the economic development of the country.

## Introduction

Individuals living primarily in rural areas of low-income countries commonly harbor multiple parasitic infections, including infection with multiple helminth species [1],[2],[3],[4],[5],[6],[7]. In particular, intestinal schistosomiasis and polyparasitic soil-transmitted helminths (STHs) infections constitute major public health problems in sub-Saharan Africa [5],[7],[8]. Despite the considerable attention in epidemiological literature to the profile of the aforementioned infections, there are very few human studies that have examined the morbidity implications of polyparasitism [9],[10],[11]. Investigating the implications of polyparasitism morbidity is particularly relevant for healthcare providers in many developing countries where they must decide screening and treatment strategies in resource-limited settings [10].

The United Nations' fifth report on world nutrition emphasized that malnutrition is the largest contributor to ill-health in the world and that diet-related risk factors for chronic disease are responsible for a large share of the burden of disease in low mortality developing countries [12]. Furthermore, this same report underscored that the effect of such malnutrition is exacerbated by the 4 to 5 billion individuals in the developing world who simultaneously suffer from iron deficiency and its related form of anemia, whilst it also highlighted the high prevalence of anemia throughout the developing world.

The link between hookworm infection and anaemia is well known, and the mechanism of effect through intestinal blood loss has been described [13],[14],[15],[16],[17],[18]. Recent large scale studies have suggested links between heavy intensities of *Schistosoma mansoni* infections (the intestinal type of schistosomiasis mainly found in sub-Saharan Africa) with anaemia and lowered haemoglobin counts [19],[20],[21]. The mechanisms underlying *S. mansoni* associated anaemia are likely multifactorial (e.g., iron deficiency due to extra-corporeal loss, splenic sequestration, autoimmune haemolysis and anaemia of inflammation) and have also been documented [22],[23]. Moderate or high intensities of *Trichuris trichiura* are also associated with higher risks of anaemia in the presence of other STHs [24], while the impact of *Ascaris lumbricoides* on anaemia is less clear [10].

Different types of helminth infection may affect nutritional status in different ways (e.g., nutrient absorption, and degree of mucosal damage) [25]. Previous studies indicated various mechanisms through which hookworm, *S. mansoni*, *T. trichiura* and *A. lumbricoides* infections might alter nutritional status [11],[22],[26],[27]. Several studies have found positive associations between malnutrition and the aforementioned intestinal parasites, but they have always limited their focus to single helminth species rather than looking at combinations of helminth species present [28],[29],[30],[31]. In addition, Ezeamama and colleagues [9] have emphasized the lack, and at the same time the need for, epidemiological studies that examine the effect of polyparasite infections at various intensities in a range of morbidities.

In the present study, we have used uniquely detailed data from the Rwandan national Neglected Tropical Disease (NTD) control programme in order to refine and understand the functional significance of polyparasite infections in anaemia and undernutrition in mainly school aged children from two districts in Northern Rwanda. The objectives of this study were to examine the distribution and the intensities of such polyparasite infections as well as to elucidate whether if individuals concurrently infected with multiple helminth species have measurably increased odds of being anaemic and/or undernourished.

## Methods

### Ethics statement

Ethical approval for Monitoring & Evaluation (M & E) surveys was obtained from the Rwandan National Ethical Committee and Columbia University's International Review Board. The aim of the survey was explained to the participants, their parents, guardians and teachers before data collection. Moreover, only children who had completed their assent form and presented a consent form signed by their parents were entered in these surveys.

### Control programme, study sites, population, sampling, and design

Rwanda is a landlocked country in the Great Lakes region of east-central Africa, bordered by Uganda, Burundi, the Democratic Republic of the Congo and Tanzania. It is one of the smallest countries of Africa (26, 338 km^2^), but is home to approximately 10.1 million people thus supporting the densest population in continental Africa, with most of the population engaged in subsistence agriculture. A verdant country of fertile and hilly terrain with altitudes varying from 950 m to 4519 m, the small republic bears the title “Land of a Thousand Hills”.

The Rwanda MoH through the Centre for Treatment and Research on AIDS, Malaria, Tuberculosis and Other Epidemics (TRAC Plus) - a centre for infectious disease control - was charged with planning and implementing data collection with the assistance from the National Reference Laboratory and the Access project.

For the M & E survey, schools in both districts were randomly selected from three sample frames to allow the programme to be evaluated in 2 low-, 2 medium- and 2 high-schistosomiasis prevalence schools. More precisely these sample frames were defined as follows:

- one school located less than 1 km away from each of the Lakes Ruhondo and Burera- this was aimed to represent a high schistosomiasis prevalence school in each of the 2 districts
- one school located between 2 km and 3 km away from each of the Lakes Ruhondo and Burera- this was aimed to represent a medium schistosomiasis prevalence school in each of the 2 districts
- one school located between 4 km and 5 km away from each of the Lakes Ruhondo and Burera- this was aimed to represent a low schistosomiasis prevalence school in each of the 2 districts

It should be noted that the aforementioned lakes are located in different districts and they were selected on the basis that distance to the lakeshore has been proven useful to screen schools in the greater region [32]. The required sample sizes for children were calculated based on schistosomiasis prevalence/intensity data from schools in various African countries with similar age ranges assuming expected reductions in *S. mansoni* intensities over two annual treatments through EpiSchisto software (http://www.schoolsandhealth.org/epidynamics.htm); more technical details have been described elsewhere [33] and hence they are not repeated here. In addition, 120 adults were randomly selected in two villages from the two aforementioned districts, which were located less than 1 km away from each of the lakes. This adult sub-group was included with the aim of monitoring the future impact of Mass Drug Administration (MDA) on *S. mansoni*-related hepatic fibrosis, where highest morbidity/symptomology tends to be displayed in this older age group. However, for the purposes of the current analyses, we decided to include data from individuals of up to 20 years old, thereby inclusive of the end of the growing period for late maturers.

These data were collected during February to April in 2008, based on results of mapping surveys in 2007 (data not presented here) with the aim to determine pre-treatment levels of the infection status and some clinical indicators.

### Infection intensity

The parasite burden was determined by duplicate examination from different microscopists of one stool specimen, at the same time, from each study participant for the presence of *S. mansoni*, *T. trichiura*, *A. lumbricoides* and hookworm (*Ancylostoma duodenale*) by the Kato-Katz method. This was due to logistical and financial reasons and can be justified within the scale of a large-scale control programme, although we are fully aware that replicate stool samples over several days are ideally required to accurately estimate intensity of schistosomiasis and STH. The mean number of eggs per gram (EPG) of stool for each parasite was used to define infections of low and moderate/high (M+) intensity in accordance with WHO-established intensity cutoff values for *S. mansoni*, *T. trichiura*, hookworm and *A. lumbricoides* infections.

### Primary determinant: parasite infection profiles

Parasite infection profiles were based on infection status of the study participants; these parasite infection profiles were created using a similar technique developed in a study conducted in rice-farming villages in Leyte, The Philippines [9].

Given possible concurrent infection by up to four parasites at one of three potential intensity levels (none, low, or M+) for each species, there were 3^4^ = 81 possible unique categories of polyparasite infections. A total of 47 of the 81 categories were found in the current Rwandese study population. The sub profiles were finally condensed into the following 4 infection profiles corresponding to putatively different risk levels for anaemia and undernutrition:

1. Reference profile (n = 189): no infection or infection with 1 parasite species at low intensity;
2. Polyparasite infection profile I (n = 582): concurrent infection with 2, 3, or 4 parasite species at low intensity;
3. Polyparasite infection profile II (n = 543): infection with 1 parasite species at M+ intensity and all other parasite species present at low intensity or absent;
4. Polyparasite infection profile III (n = 291): concurrent infection with at least 2 parasite species at M+ intensity and all other parasite species present at low intensity or absent. More precisely, in this polyparasite infection profile 6 children had concurrent infection with 3 or 4 parasites at M+ intensity.

### Morbidity indicators

Heights were measured with height poles which had a fixed head board and can thus be considered comparable to that of the NHANES stadiometer (http://www.cdc.gov/nchs/products/elec_prods/subject/video.htm). More precisely, the stature meter was placed to the floor and for each individual the tape was pulled up until zero reached the red line. The upper part of the pole was then firmly and accurately attached to the wall and fixed with screws. Finally the meter was pulled down onto the head of individual to get the measurement. Weights were measured with electronic balances. Children were asked to remove their shoes and all heavy clothes if they wore any and this was done in the morning by the survey team. All persons performing these measurements were fully trained and experienced in the use of these protocols, and the same staffs were used throughout to ensure standardization.

Finger prick blood samples were also obtained from each individual, sufficient for accurate Hb measurement using a Hemocue photometer [34]. Indices of the anthropometric status of the studied children were based on the 2000 growth reference curves designed by the Centre for Disease Control (CDC) as this population more closely resembles those in countries like Rwanda since it includes both human milk and formula-fed infants; these were computed using the Nutstat program within Epi Info V 3.4. The fact that the 2000 CDC growth charts consist of sex specific charts for infants, birth to age 36 months (length-for-age, weight-for-length, weight-for-age, and head circumference-for-age) and older children, 2 to 20 years (stature-for-age, weight-for-age and Body Mass Index (BMI)-for-age) led us also to the decision of excluding data of individuals more than 20 years old. Low Body Mass Index is considered an indicator of acute under-nutrition (thinness or wasting) and is generally associated with failure to gain weight or a loss of weight [35]. The Z-score cut-off point recommended by WHO, CDC, and others to classify low anthropometric levels is 2 Standard Deviation (SD) units below the reference median for this specific index. A cut-off of -2 BMI Z-scores was calculated to classify underweight individuals. The z-scores of height-for-age that were less than 2 SD below the reference median served to define stunted individuals.

### Statistical methods

In order to examine the adjusted odds ratios (ORs) of anaemia, wasting and stunting, we tested a range of different approaches of statistical modeling to correctly identify individuals who have had higher morbidity as assessed from the outcomes aforementioned. Because the modeling of the between school variation through random effects logistic regressions did not prove appropriate for the statistical analysis of our data, we also employed the Generalized Estimating Equations (GEE) approach whenever this was analytically possible. If the GEE algorithm did not converge, we used conventional logistic regression models. The GEE method does not explicitly model between-cluster variation; instead it focuses on and it estimates its counterpart, the within-cluster similarity of the residuals; it then uses this estimated correlation to reestimate the regression parameters and to calculate standard errors which are reasonably accurate and hence lead to the generation of confidence intervals with the correct coverage rates [36]. Data management and statistical analyses were performed using SAS V9 (SAS Institute Inc., Cary, NC, USA).

For all the odds ratios studied, we fitted the random effects logistic regression models by using PROC NLMIXED while we employed the GEE method by using PROC GENMOD. Particularly for the odds of anaemia, we have included as explanatory variable the parasite infection profiles I-III (as defined in the previous section); we also consider the nutritional status as defined by stunting as an effect modifier. We therefore display estimates with and without considering the effect of stunting; we also included the interaction term of stunting with the parasite infection profiles if the change in deviances between relevant nested models was significant at the 5% significance level. Similarly, when we modeled the odds of wasting and stunting respectively, we have included as explanatory variable the parasite infection profiles I-III while we consider anaemia status as an effect modifier. Potential confounders of the relationships between anaemia stunting, wasting and helminth infection were decided to be included in light of known confounders of these associations based on published literature [11],[20],[21],[37],[38],[39] and these were the categories of age, sex, and the district where study participants were living in.

Mean Hb concentration of different groups of individuals recruited in the current study were also initially examined through a random effects at the school level multivariate linear regression model by using PROC MIXED. Likelihood ratio tests indicated that these random effects were not significant and thus it was finally decided to omit them. However, because of the non-independence found in our data we finally decided to employ the GEE method by using PROC GENMOD. We tested as explanatory variables the categories of age, sex, district and parasite infection profiles I-III. We also tested the two-way interaction terms of parasite infection profiles I-III with district and stunting and retained them in the model if the change in deviances between the relevant nested models was significant at the 5% significance level.

Covariates in all aforementioned multivariable models with p<0.05 were considered significantly associated with outcomes.

## Results

A total of 1605 children and adolescents were recruited, for a participation rate of 88%, and provided complete parasitologic and anthropometric data. They were aged 5 to 20 years old, with a median age of 10 years and 47.7% of the recruited individuals were male. The observed prevalences of wasting, stunting and anaemia were respectively estimated as following: 8.1% (95% CI: 6.8 to 9.4), 38.5% (36.1 to 40.9) and 4.9% (95% CI: 3.9 to 6.1). The mean observed Hb concentration was estimated to be 13.8 g/dL (95% CI: 13.7 to 13.8).

Table 1 contains the characteristics of the study population by subprofile classification infection category. The most prevalent co-infections were those of low intensity of *A. lumbricoides* and *T. trichiura* (21.2%).

**Table 1:** Characteristics of the study population.

Subprofile classification, infection category	Children No (%)	Mean hemoglobin level, g/dL	Individuals with anemia^a^ No (%)^b^	Stunted Individuals^c^ No (%)^d^	Wasted Individuals^e^ No (%)^f^
**Subprofile 0: 0 or 1 L infection (n = 189)**					
0 infections	31 (1.93)	13.72	1 (3.23)	12 (38.71)	1 (3.23)
*S. mansoni*	4 (0.25)	14.20	0 (0.00)	2 (50.00)	0 (0.00)
Hookworm	12 (0.75)	13.58	1 (8.33)	4 (33.33)	1 (8.33)
*T. trichiura*	97 (6.04)	13.64	5 (5.15)	34 (35.05)	1 (1.03)
*A. lumbricoides*	45 (2.80)	13.81	4 (8.89)	16 (35.56)	5 (11.11)
**Subprofile 1: 2 L infections (n = 404)**					
*S. mansoni* and Hookworm	2 (0.12)	14.30	0 (0.00)	2 (100.00)	0 (0.00)
*S. mansoni* and *A. lumbricoides*	10 (0.62)	14.26	0 (0.00)	3 (30.00)	2 (20.00)
*S. mansoni* and *T. trichiura*	14 (0.87)	13.99	0 (0.00)	7 (50.00)	2 (14.29)
*T. trichiura* and Hookworm	28 (1.74)	14.32	2 (7.14)	12 (42.86)	2 (7.14)
*A. lumbricoides* and Hookworm	10 (0.62)	13.75	0 (0.00)	6 (60.00)	1 (10.00)
*A. lumbricoides* and *T. trichiura*	340 (21.18)	13.74	16 (4.71)	121 (35.59)	26 (7.65)
**Subprofile 2: 3 L infections (n = 157)**					
*S. mansoni*, Hookworm and *T. trichiura*	4 (0.25)	15.25	0 (0.00)	1 (25.00)	0 (0.00)
*S. mansoni*, Hookworm and *A. lumbricoides*	2 (0.12)	13.90	0 (0.00)	0 (0.00)	0 (0.00)
*S. mansoni*, *A. lumbricoides* and *T. trichiura*	54 (3.36)	14.01	2 (3.70)	20 (37.04)	2 (3.70)
*A. lumbricoides*, Hookworm and *T. trichiura*	97 (6.04)	13.91	6 (6.19)	48 (49.48)	2 (2.06)
**Subprofile 3: 4 L infections (n = 21)**					
*A. lumbricoides*, Hookworm, *S. mansoni* and *T. trichiura*	21 (1.31)	14.16	1 (4.76)	9 (42.86)	2 (9.52)
**Subprofile 4: 1 M+ infection (n = 25)**					
*S. mansoni*	2 (0.12)	14.50	0 (0.00)	1 (50.00)	0 (0.00)
*T. trichiura*	11 (0.69)	13.81	0 (0.00)	4 (36.36)	2 (18.18)
*A. lumbricoides*	12 (0.75)	13.62	0 (0.00)	6 (50.00)	1 (8.33)
**Subprofile 5: 1 M+ infection and 1 L infections (n = 337)**					
*S. mansoni* (M+) and Hookworm (L)	2 (0.12)	15.75	0 (0.00)	2 (100.00)	0 (0.00)
*S. mansoni* (M+) and *T. trichiura* (L)	2 (0.12)	14.20	0 (0.00)	1 (33.33)	0 (0.00)
*S. mansoni* (M+) and *A. lumbricoides* (L)	4 (0.25)	14.23	0 (0.00)	1 (25.00)	0 (0.00)
*T. trichiura* ( M+) and *A. lumbricoides* (L)	47 (2.93)	13.57	2 (4.26)	12 (25.53)	7 (14.89)
*T. trichiura* ( M+) and *S. mansoni* (L)	1 (0.06)	14.10	0 (0.00)	1 (100.00)	1 (100.00)
*T. trichiura* ( M+) and Hookworm (L)	1 (0.06)	14.70	0 (0.00)	0 (0.00)	0 (0.00)
*A. lumbricoides* (M+) and Hookworm (L)	7 (0.44)	14.26	0 (0.00)	1 (14.29)	0 (0.00)
*A. lumbricoides* (M+) and *T. trichiura* (L)	273 (17.01)	13.58	16 (5.86)	102 (37.36)	24 (8.79)
**Subprofile 6: 1 M+ infection and 2 L infections (n = 149)**					
*S. mansoni* (M+), Hookworm (L) and *T. trichiura* (L)	5 (0.31)	14.50	0 (0.00)	2 (40.00)	0 (0.00)
*S. mansoni* (M+), *A. lumbricoides* (L) and *T. trichiura* (L)	18 (1.12)	14.50	1 (5.56)	9 (50.00)	2 (11.11)
*T. trichiura* ( M+), Hookworm (L) and *A. lumbricoides* (L)	15 (0.93)	14.09	0 (0.00)	9 (60.00)	1 (6.67)
*T. trichiura* ( M+), *S.mansoni* (L) and *A. lumbricoides* (L)	4 (0.25)	13.40	0 (0.00)	2 (50.00)	1 (25.00)
*T. trichiura* ( M+), *S.mansoni* (L) and Hookworm (L)	1 (0.06)	10.40	1 (100.00)	0 (0.00)	0 (0.00)
*A. lumbricoides* (M+), Hookworm (L) and *T. trichiura* (L)	81 (5.05)	13.84	5 (6.17)	37 (45.68)	5 (6.17)
*A. lumbricoides* (M+), *S.mansoni* (L) and *T. trichiura* (L)	25 (1.56)	13.96	3 (12.00)	14 (56.00)	2 (8.00)
**Subprofile 7: 1 M+ infection and 3 L infections (n = 32)**					
*S. mansoni* (M+), Hookworm (L), *A. lumbricoides* (L) and *T. trichiura* (L)	20 (1.25)	13.92	1 (5.00)	12 (60.00)	0 (0.00)
*A. lumbricoides* (M+), *S.mansoni* (L), Hookworm (L) and *T. trichiura* (L)	12 (0.75)	14.33	0 (0.00)	3 (25.00)	1 (8.33)
**Subprofile 8: 2 M+ infections (n = 201)**					
*A. lumbricoides* (M+) and *T. trichiura* (M+)	201 (12.52)	13.63	10 (4.98)	74 (36.82)	33 (16.42)
**Subprofile 9: 2 M+ and 1 L infections (n = 64)**					
*S. mansoni* (M+), *T. trichiura* (M+) and *A. lumbricoides* (L)	2 (0.12)	12.05	1 (50.00)	1 (50.00)	0 (0.00)
*S. mansoni* (M+), *A. lumbricoides* (M+) and *T. trichiura* (L)	17 (1.06)	14.29	1 (5.26)	5 (29.41)	0 (0.00)
*A. lumbricoides* (M+), *T. trichiura* (M+) and *S. mansoni* (L)	15 (0.93)	13.42	0 (0.00)	7 (46.67)	2 (13.33)
*A. lumbricoides* (M+), *T. trichiura* (M+) and Hookworm (L)	29 (1.81)	13.27	1 (3.45)	8 (27.59)	1 (3.45)
*A. lumbricoides* (M+), *S. mansoni* (M+) and *T. trichiura* (L)	1 (0.06)	14.60	0 (0.00)	0 (0.00)	
**Subprofile 10: 2 M+ and 2 L infections (n = 20)**					
*S. mansoni* (M+), *T. trichiura* (M+), Hookworm (L) and *A. lumbricoides* (L)	2 (0.12)	14.65	0 (0.00)	1 (50.00)	0 (0.00)
*S. mansoni* (M+), *A. lumbricoides* (M+), *T. trichiura* (L) and Hookworm (L)	7 (0.44)	14.49	0 (0.00)	0 (0.00)	0 (0.00)
*A. lumbricoides* (M+), *T. trichiura* (M+), Hookworm (L) and *S. mansoni* (L)	11 (0.69)	14.35	0 (0.00)	6 (54.55)	0 (0.00)
Subprofile 11: 3 M+ infections or 3 M+ and 1 L infections (n = 6)					
*S. mansoni* (M+), *A. lumbricoides* (M+), *T. trichiura* (M+)	2 (0.12)	11.80	1 (50.00)	1 (50.00)	0 (0.00)
*S. mansoni* (M+), *A. lumbricoides* (M+), *T. trichiura* (M+) and Hookworm (L)	4 (0.25)	12.85	0 (0.00)	0 (0.00)	0 (0.00)

a Anaemia was defined for all tables displayed (according to WHO guidelines), as Hb less than 11.5 g/dL for children from 5 to 11 years old and for children between 12 and 14 years old as Hb less than 12.0 g/dL. For individuals aged more than 14 years old, anaemia was defined as Hb less 12.0 g/dL for females and Hb less than 13.0 g/dL for males.

b Percentages in this column denote percentages of anaemic within each specific subprofile classification/infection category.

c Stunting was defined as height for age z-score (HAZ) less than -2.

d Percentages in this column denote percentages of stunted within each specific subprofile classification/infection category.

e Wasting was defined as body mass index z-score (BMIZ) less than -2.

f Percentages in this column denote percentages of wasted within each specific subprofile classification/infection category.

The adjusted ORs of anaemia from the GEE multivariate logistic regression models are presented in Table 2. Deviance tests indicated ‘Model 3’ as the most appropriate one; this model shows that individuals who are stunted are almost 1.5 times more likely than non-stunted to be anaemic (OR: 1.6, P = 0.041). Children of 11–13 years old were significantly less likely than children of 5–7 years old to be anaemic (OR = 0.572, P = 0.026). In addition, males are almost twice more likely to be anaemic compared to females (OR: 1.9, P = 0.024). Neither the interaction terms of stunting or district with the parasite infection profiles nor any other examined variable here were found to be significant factors for the odds of being anaemic.

**Table 2:** Adjusted ORs from GEE multivariate logistic regression model of anaemia (n = 1605).

Variable	Categories	Adjusted ORs (95% CI)○	p-values
Model 1†, Deviance = 623.193, DF = 1596			
*Age*	5–7 years	1	
	8–10 years	0.647 (0.381–1.099)	0.107
	11–13 years	0.631 (0.385–1.034)	0.068
	14–17 years	0.759 (0.408–1.411)	0.383
	18–20 years	1.629 (0.732–3.622)	0.232
*Sex*	Female	1	
	Male	2.022 (1.145–3.569)	0.015
*Polyparasite infection profile* *	Reference	1	
	I	0.793 (0.499–1.258)	0.324
	II	0.877 (0.605–1.271)	0.489
	III	0.733 (0.445–1.209)	0.224
Model 2‡, Deviance = 622.259, DF = 1595			
*Age*	5–7 years	1	
	8–10 years	0.640 (0.373–1.099)	0.106
	11–13 years	0.621 (0.383–1.006)	0.053
	14–17 years	0.747 (0.411–1.357)	0.338
	18–20 years	1.682 (0.662–4.276)	0.275
*Sex*	Female	1	
	Male	2.018 (1.140–3.571)	0.016
*Polyparasite infection profile*	Reference	1	
	I	0.799 (0.495–1.290)	0.359
	II	0.855 (0.614–1.191)	0.354
	III	0.694 (0.436–1.107)	0.101
*District*	Musanze	1	
	Burera	1.258 (0.826–1.916)	0.650
Model 3◊, Deviance = 619.540, DF = 1595			
*Age*	5–7 years	1	
	8–10 years	0.619 (0.367–1.043)	0.072
	11–13 years	0.572 (0.350–0.935)	0.026
	14–17 years	0.715 (0.405–1.263)	0.248
	18–20 years	1.674 (0.784–3.574)	0.183
*Sex*	Female	1	
	Male	1.883 (1.086–3.265)	0.024
*Polyparasite infection profile*	Reference	1	
	I	0.788 (0.501–1.238)	0.300
	II	0.863 (0.607–1.228)	0.413
	III	0.739 (0.449–1.217)	0.235
*Stunting*	Non stunted	1	
	Stunted	1.590 (1.018–2.484)	0.041

**○:** 95% CIs are based on empirical standard errors.

**†:** Model 1 included as explanatory variables the categories of age, sex and polyparasite infection profiles.

*: Definitions for polyparasite profiles have been described in the ‘Methods’ section.

**‡:** Model 2 included as explanatory variables the categories of age, sex, polyparasite infection profiles and district.

**◊:** Model 3 included as explanatory variables the categories of age, sex, polyparasite infection profiles, and stunting.

GEE did not converge for the modeling of the odds of being wasted and this is most likely to be explicable by the fact that there was not sufficient information in order to estimate the binomial probability structure by taking into account the intra-subject correlation. Consequently, we used Maximum Likelihood (ML) and the results of such multivariate logistic regression models for the odds of being wasted are presented in Table 3. Deviance tests as well as Akaike's information criterion (AIC) indicated ‘Model 2’as the best one among the tested models. This model shows that only children of 11–13 years old were significantly more likely than the younger children (age group: 5–7 years old) to be wasted (OR = 1.8, P = 0.033). Furthermore, study participants from Burera district were significantly more likely to be wasted when compared with study participants from Musanze district (OR = 3.3, P<0.001). It is noteworthy that children with concurrent infection of at least 2 parasite species at M+ intensity - that is, those with polyparasite infection profiles III - were almost twice marginally significantly more likely to be wasted than children with the reference polyparasite infection profile (OR = 2.2, P = 0.054). Neither the interaction terms of anaemia status or district with the parasite infection profiles nor any other examined variable here, were found to be significant factors for the odds of being wasted.

**Table 3:** Adjusted odds ratios from ML multivariate logistic regression model of wasting (n = 1605).

Variable	Categories	Adjusted ORs (95% CI)	p-values
Model 1†, Deviance = 880.568, AIC = 898.568, DF = 1596			
*Age*	5–7 years	1	
	8–10 years	0.995 (0.593–1.671)	0.985
	11–13 years	1.831 (1.061–3.161)	0.030
	14–17 years	0.986 (0.573–1.697)	0.959
	18–20 years	NA[Table-fn nt113]	NA
*Sex*	Female	1	
	Male	0.946 (0.658–1.359)	0.763
*Polyparasite infection profile* *	Reference	1	
	I	1.575 (0.721–3.442)	0.255
	II	2.093 (0.969–4.522)	0.060
	III	3.081 (1.396–6.800)	0.005
Model 2‡, Deviance = 846.862, AIC = 866.862, DF = 1595			
*Age*	5–7 years	1	
	8–10 years	1.028 (0.609–1.736)	0.917
	11–13 years	1.824 (1.050–3.170)	0.033
	14–17 years	0.975 (0.564–1.684)	0.927
	18–20 years	NA	NA
*Sex*	Female	1	
	Male	0.973 (0.674–1.403)	0.882
*Polyparasite infection profile*	Reference	1	
	I	1.617 (0.735–3.556)	0.232
	II	1.779 (0.817–3.872)	0.147
	III	2.206 (0.988–4.928)	0.054
*District*	Musanze	1	
	Burera	3.264 (2.136–4.990)	<0.001
Model 3◊, Deviance = 846.366, AIC = 868.366, DF = 1594			
*Age*	5–7 years	1	
	8–10 years	1.023 (0.606–1.727)	0.932
	11–13 years	1.808 (1.040–3.144)	0.036
	14–17 years	0.968 (0.560–1.673)	0.907
	18–20 years	NA	NA
*Sex*	Female	1	
	Male	0.967 (0.670–1.395)	0.858
*Polyparasite infection profile*	Reference	1	
	I	1.604 (0.729–3.530)	0.240
	II	1.775 (0.816–3.865)	0.148
	III	2.195 (0.983–4.905)	0.055
*District*	Musanze	1	
	Burera	3.271 (2.140–5.000)	<0.001
*Anaemia*	Non Anaemic	1	
	Anaemic	0.724 (0.283–1.849)	0.499

**†:** Model 1 included as explanatory variables the categories of age, sex and polyparasite infection profiles.

▪•.NA stands for ‘Not Available’ as there were no individuals of 18–20 years old who were wasted and thus the model did not provide any estimates for thsi specific category.

*: Definitions for polyparasite profiles have been described in the ‘Methods’ section.

**‡:** Model 2 included as explanatory variables the categories of age, sex, polyparasite infection profiles and district.

**◊:** Model 3 included as explanatory variables the categories of age, sex, polyparasite infection profiles, and district and anaemia status.

Table 4 contains the results from the GEE multivariate logistic regression models for the odds of being stunted. Deviance tests indicated ‘Model 3’ as the most appropriate one; this model shows those children of 11–17 years old to have significant positive ORs if compared with the youngest age group examined here (i.e. 5–7 years old), (more specifically, 11–13 years old: OR = 2.4, P = 0.001; 14–17 years old: OR = 1.4, P = 0.044). However, adolescents of 18–20 years old were significantly less likely than the youngest age group to be stunted (OR = 0.4, P = 0.003). Male individuals were significantly more likely than females to be stunted (OR = 1.9, P<0.001). Study participants from Burera district were significantly less likely to be stunted than the study participants from Musanze district (OR = 0.4, P<0.001). Anaemic study participants were significantly more likely than non anaemic to be stunted (OR = 1.7, P = 0.020). Neither the interaction terms of anaemia status or district with the parasite infection profiles nor any other examined variable here were found to be significant factors for the odds of being stunted.

**Table 4:** Adjusted odds ratios from GEE multivariate logistic regression model of stunting (n = 1605).

Variable	Categories	Adjusted ORs (95% CI)○	p-values
Model 1†, Deviance = 2073.436, DF = 1596			
*Age*	5–7 years	1	
	8–10 years	1.390 (0.937–2.062)	0.102
	11–13 years	2.237 (1.370–3.654)	0.001
	14–17 years	1.337 (0.953–1.875)	0.092
	18–20 years	0.360 (0.143–0.909)	0.031
*Sex*	Female	1	
	Male	1.904 (1.648–2.200)	<0.001
*Polyparasite infection profile* *	Reference	1	
	I	1.030 (0.742–1.431)	0.858
	II	1.249 (0.849–1.837)	0.259
	III	1.136 (0.642–2.010)	0.662
Model 2‡, Deviance = 2016.559, DF = 1595			
*Age*	5–7 years	1	
	8–10 years	1.450 (0.963–2.182)	0.075
	11–13 years	2.354 (1.414–3.919)	0.001
	14–17 years	1.403 (0.998–1.972)	0.051
	18–20 years	0.405 (0.214–0.769)	0.006
*Sex*	Female	1	
	Male	1.973 (1.700–2.289)	<0.001
*Polyparasite infection profile*	Reference	1	
	I	1.043 (0.747–1.456)	0.806
	II	1.280 (0.872–1.880)	0.208
	III	1.189 (0.660–2.142)	0.565
*District*	Musanze	1	
	Burera	0.427 (0.309–0.588)	<0.001
Model 3◊, Deviance = 2012.140, DF = 1594			
*Age*	5–7 years	1	
	8–10 years	1.465 (0.973–2.206)	0.068
	11–13 years	2.384 (1.428–3.981)	0.001
	14–17 years	1.412 (1.009–1.977)	0.044
	18–20 years	0.397 (0.214–0.735)	0.003
*Sex*	Female	1	
	Male	1.942 (1.692–2.228)	<0.001
*Polyparasite infection profile*	Reference	1	
	I	1.049 (0.755–1.458)	0.775
	II	1.286 (0.884–1.871)	0.189
	III	1.201 (0.672–2.146)	0.536
*District*	Musanze	1	
	Burera	0.424 (0.307–0.585)	<0.001
*Anaemia*	Non Anaemic	1	
	Anaemic	1.671 (1.086–2.572)	0.020

**○:** 95% CIs are based on empirical standard errors.

**†:** Model 1 included as explanatory variables the categories of age, sex and polyparasite infection profiles.

*: Definitions for polyparasite profiles have been described in the ‘Methods’ section.

**‡:** Model 2 included as explanatory variables the categories of age, sex, polyparasite infection profiles and district.

**◊:** Model 3 included as explanatory variables the categories of age, sex, polyparasite infection profiles, and district and anaemia status.

Finally, Table 5 contains the results from the GEE linear regression model for the mean Hb concentration and the mean differences in different groups of the study population here. Deviance tests indicated ‘Model 3’ as the most appropriate one; this model shows that on average Hb concentration in the study population was 13.109 g/dL (95% CI: 12.904–13.314). All different categories of age yielded significant associations with increased Hb levels compared to the youngest age group examined here (i.e., 5–7 years old). Study participants who were stunted when compared to non-stunted had significantly lower Hb counts by 0.270 g/dL, respectively (P<0.001).

**Table 5:** Estimated differences in mean Hb concentration at baseline for the effects of selected explanatory variables from a GEE linear regression model (n = 1605).

Variable	Categories	Adjusted mean differences (95% CI) ○	p-values
Model 1†, Deviance = 2385.795, DF = 1596			
*Age*	*Intercept* (5–7 years, female, reference polyparasite profile)	13.052 (12.859–13.246)	<0.001
	8–10 years	0.473 (0.391–0.556)	<0.001
	11–13 years	0.845 (0.679–1.012)	<0.001
	14–17 years	1.342 (1.113–1.571)	<0.001
	18–20 years	1.628 (1.137–2.120)	<0.001
*Sex*	Male	−0.100 (−0.227–0.027)	0.124
Polyparasite infection profile^*^	I	0.093 (−0.131–0.317)	0.418
	II	0.079 (−0.167–0.325)	0.530
	III	0.052 (−0.145–0.248)	0.605
Model 2‡, Deviance = 2381.176, DF = 1595			
*Age*	*Intercept* (5–7 years, female, reference polyparasite profile, Musanze)	13.098 (12.877–13.319)	<0.001
	8–10 years	0.471 (0.392–0.550)	<0.001
	11–13 years	0.845 (0.681–1.010)	<0.001
	14–17 years	1.342 (1.115–1.568)	<0.001
	18–20 years	1.631 (1.154–2.109)	<0.001
*Sex*	Male	−0.099 (−0.229–0.031)	0.135
*Polyparasite infection profile*	I	0.090 (−0.135–0.315)	0.434
	II	0.081 (−0.169–0.330)	0.527
	III	0.057 (−0.154–0.268)	0.597
*District*	Burera	−0.091 (−0.352–0.171)	0.497
Model 3◊, Deviance = 2361.793, DF = 1595			
*Age*	*Intercept* (5–7 years, female, reference polyparasite profile, non stunted)	13.109 (12.904–13.314)	<0.001
	8–10 years	0.494 (0.426–0.561)	<0.001
	11–13 years	0.896 (0.755–1.037)	<0.001
	14–17 years	1.360 (1.142–1.577)	<0.001
	18–20 years	1.583 (1.081–2.085)	<0.001
*Sex*	Male	−0.060 (−0.181–0.061)	0.330
*Polyparasite infection profile*	I	0.096 (−0.139–0.330)	0.424
	II	0.092 (−0.155–0.340)	0.464
	III	0.060 (−0.155–0.274)	0.587
*Stunting*	Stunted	−0.270 (−0.336–−0.205)	<0.001

○95 % CIs are based on empirical standard errors.

**†:** Model 1 included as explanatory variables the categories of age, sex and polyparasite infection profiles.

*Definitions for polyparasite profiles have been described in the ‘Methods’ section.

**‡:** Model 2 included as explanatory variables the categories of age, sex, polyparasite infection profiles and district.

**◊:** Model 3 included as explanatory variables the categories of age, sex, polyparasite infection profiles, and stunting.

## Discussion

Concurrent multiple parasite infections were found to be the norm in our study population, as has been reported in studies published elsewhere [1],[2],[5],[6],[9],[10]. However, in the current study population, none of the concurrent polyparasite infections were found to be significantly associated with higher odds of anaemia, wasting, stunting nor mean lowered Hb concentration. Nevertheless, results did indicate that those study participants with concurrent infection with at least 2 parasites at M+ intensity were marginally significantly more likely to be wasted (P = 0.054) relative to those with no infection or infection with 1 parasite species at low intensity, thereby validating the impact of higher intensity infections on health [40].

Potential reasons for the general lack of association of the concurrent polyparasite infections with anaemia in the current Rwandan population might be that anaemia itself appears to be relatively uncommon in this area. One reason for the latter may relate also to the fact that malaria incidence in the two districts studied here is lower that the rest of the country, as well as to the decrease of malaria prevalence in Rwanda in general as an unpublished WHO Draft of Mid Term Evaluation Report of the Rwandan Malaria Strategic Plan 2005–2010, reveals. In addition, as Table 1 indicates, the majority of the study participants (i.e. 21.2%) had low intensities of *A. lumbricoides* and *T. trichiura* while very few of them had M+ intensities of hookworm and *S. mansoni* infections. Such a distribution is likely to have limited the power of this study – potentially making it difficult to achieve statistical significance where one existed for co-infections of M+ intensities. M+ intensities of the latter two helminth infections have been recently shown to be significant factors for anaemia in other similar epidemiological studies [20],[21], and such combined findings highlight how different factors contribute to anaemia in different parasite transmission and eco-epidemiological settings. Indeed we would recommend further similar studies in the eastern part of Rwanda where there is a higher prevalence of hookworm as shown by the STH mapping survey conducted last year by the NTD control programme (unpublished data) and malaria together with other country and epidemiological settings, to further elucidate the potential association of polyparasitism to human morbidity.

The present study also indicated children of 11–13 years old to be significantly less likely than children of 5–7 years old to be anaemic. This finding might be explained by the fact that the youngest children have recently experienced the high iron demand of early childhood. We also found males compared to females to be significantly more likely to be anaemic. A previous study has discussed that among younger children, boys are more anaemic than girls but the reasons for this remain still unknown [41]. We have also attempted to examine if the differences between the sexes in the odds of being anaemic varied by age, but when the statistical interactions of age and sex were included in the relevant model, the algorithm did not converge. Stunted children compared to non stunted were also demonstrated to be more likely to be anaemic. This result is supported by the observation that iron deficiency which leads to anaemia also contributes to poor growth while it has been demonstrated that supplementation of iron to anemic children has a positive effect on linear growth [42]. The effect of the polyparasite infection profile on anaemia was not found to vary according to stunting.

We also assessed the association between the polyparasite infection profiles and acute under-nutrition. Children with concurrent infection with at least two parasites at M+ intensity relative to those with no infection or infection were found to be marginally significantly more likely to be wasted (P = 0.054). This finding might be explained by decreased appetite experienced in those individuals who harbored two or more parasites at M+ intensity. However, significant differences in the odds of wasting observed within the two districts studied here still remain unclear as we do not think that the latter would differ in dietary patterns or socio-economic status.

Regarding chronic undernutrition and concurrent polyparasite infections, the present cross-sectional study did not find any significant association with the exception of age and anaemia status being revealed as a significant factor for stunting. Older individuals, with the exception of the age group of 18–20 years old, were found to be more likely to be stunted than the younger age group studied here (i.e. 5–7 years old). This could imply prior malnutrition in these individuals as has previously been reported in Zanzibar and Burkina Faso [43],[44]. Furthermore, the findings of decreased odds of stunting in the older age of 18–20 years old suggest compensatory growth in height for this age group and this is consistent with results from longitudinal Senegalese data [45]. Anaemic compared to non-anaemic individuals were also found to be significantly more likely to be stunted and the causal pathways for such results have been discussed in the previous paragraph. However, the effect of the polyparasite infection profile on stunting was not found to vary according to anaemia status. Finally, it should be noted that stunting - an indicator of chronic undernutrition - was the most prevalent form of undernutrition observed in this study. This has been also found by the Rwanda demographic health survey 2005 where the Northern province had the highest prevalence of severe chronic malnutrition [46]. Nevertheless, for the same reasons as mentioned above, any explanation for the significant differences in the odds of stunting observed within the two districts studied here still remain unclear.

Our investigation has some limitations. As mentioned above, due in part to the overdispersed nature of helminth infections eggs in stool and daily variation in excretion, the ideal protocol is to use replicate faecal samples over several (ideally a minimum of three) consecutive days [47]. Unfortunately due to the logistical and financial constraints inherent within the scale of such a large-scale control programme, such ideals cannot realistically be met and hence duplicate Kato Katz thick smears were taken from a single day's stool per individual instead. We are aware that such an assessment method is likely to have introduced some misclassification in the measurement of the intensities of helminth infections and consequently in the allocation of study participants to the polyparasite infection profiles. In addition, although we do recognize that anthropometric measurements should be taken according to the standardized protocols used by NHANES to develop the growth charts, some modification to these gold standard measures are necessarily within the field conditions of Mass Drug Administration. Nevertheless, we are confident that every possible precaution was employed by the Rwanda field team in order to obtain accurate and high quality reproducible data. Furthermore, we believe that is it high unlikely that the examined associations of this study were biased by unmeasured confounding factors such as socio-economic status of the study participants despite the fact that helminth infections are known to be intimately linked with poverty [48],[49],[50],[51]. The reason for this is that surveyed participants most likely would belong to the poorest populations of the country with no significant variations in their socio-economic status and thus with no effect in the examined outcomes here. Therefore overall, despite the aforementioned potential limitations, this study represents one of the few quantitative, comprehensively analyzed studies on the epidemiology of helminth infections, anaemia and undernutrition in Rwanda covering a broad age range with an extension particularly in the adolescents' years.

In conclusion the results of this study suggest that low-intensity polyparasite infections are more prevalent in Northern Rwanda, relative to high intensity polyparasitism, at least in terms of the major species of parasites under focus in the current study, and such co-infections appear not to have, alone, a great impact on anaemia and undernutrition. Consequently based on the current findings we would support the argument that sufficient chemotherapy programmes to prevent high infection intensities build up in these people even without achieving parasite eradications, should be promoted. Finally, as currently there is a move towards drugs for integrated NTDs, we would urge for similar analytical studies in order to fully evaluate risks and benefits of such initiatives in helminth endemic regions.

## Supporting Information

Alternative Language Abstract S1 Translation of the abstract into French by MAD.
(0.03 MB DOC)

## References

[1] Ashford RW, Craig PS, Oppenheimer SJ (1993). Polyparasitism on the Kenya coast. 2. Spatial heterogeneity in parasite distributions.. Ann Trop Med Parasitol.

[2] Chunge RN, Karumba N, Ouma JH, Thiongo FW, Sturrock RF (1995). Polyparasitism in two rural communities with endemic *Schistosoma mansoni* infection in Machakos District, Kenya.. J Trop Med Hyg.

[3] de Cassia Ribeiro Silva R, Barreto ML, Assis AM, de Santana ML, Parraga IM (2007). The relative influence of polyparasitism, environment, and host factors on schistosome infection.. Am J Trop Med Hyg.

[4] Drake LJ, Bundy DA (2001). Multiple helminth infections in children: impact and control.. Parasitology.

[5] Keiser J, N'Goran EK, Traore M, Lohourignon KL, Singer BH (2002). Polyparasitism with Schistosoma mansoni, geohelminths, and intestinal protozoa in rural Cote d'Ivoire.. J Parasitol.

[6] Tchuem Tchuente LA, Behnke JM, Gilbert FS, Southgate VR, Vercruysse J (2003). Polyparasitism with *Schistosoma haematobium* and soil-transmitted helminth infections among school children in Loum, Cameroon.. Trop Med Int Health.

[7] Thiong'o FW, Luoba A, Ouma JH (2001). Intestinal helminths and schistosomiasis among school children in a rural district in Kenya.. East Afr Med J.

[8] Rietveld E, Vetter JC, Stilma JS (1987). Concurrent parasitic infections among patients with onchocerciasis and controls in Sierra Leone, West Africa.. Doc Ophthalmol.

[9] Ezeamama AE, Friedman JF, Olveda RM, Acosta LP, Kurtis JD (2005). Functional significance of low-intensity polyparasite helminth infections in anemia.. J Infect Dis.

[10] Ezeamama AE, McGarvey ST, Acosta LP, Zierler S, Manalo DL (2008). The Synergistic Effect of Concomitant Schistosomiasis, Hookworm, and Trichuris Infections on Children's Anemia Burden.. PLoS Negl Trop Dis.

[11] Jardim-Botelho A, Brooker S, Geiger SM, Fleming F, Souza Lopes AC (2008). Age patterns in undernutrition and helminth infection in a rural area of Brazil: associations with ascariasis and hookworm.. Trop Med Int Health.

[12] UN SCN (United Nations System Sub-Committee on Nutrition) (2004). 5th Report on the World Nutrition Situation: Nutrition for Improved Development Outcomes.

[13] Brooker S, Peshu N, Warn PA, Mosobo M, Guyatt HL (1999). The epidemiology of hookworm infection and its contribution to anaemia among pre-school children on the Kenyan coast.. Trans R Soc Trop Med Hyg.

[14] Changhua L, Xiaorong Z, Dongchuan Q, Shuhua X, Hotez PJ (1999). Epidemiology of human hookworm infections among adult villagers in Hejiang and Santai Counties, Sichuan Province, China.. Acta Trop.

[15] Crompton DW, Whitehead RR (1993). Hookworm infections and human iron metabolism.. Parasitology.

[16] Olsen A, Magnussen P, Ouma JH, Andreassen J, Friis H (1998). The contribution of hookworm and other parasitic infections to haemoglobin and iron status among children and adults in western Kenya.. Trans R Soc Trop Med Hyg.

[17] Stoltzfus RJ, Albonico M, Chwaya HM, Savioli L, Tielsch J (1996). Hemoquant determination of hookworm-related blood loss and its role in iron deficiency in African children.. Am J Trop Med Hyg.

[18] Stoltzfus RJ, Chwaya HM, Tielsch JM, Schulze KJ, Albonico M (1997). Epidemiology of iron deficiency anemia in Zanzibari schoolchildren: the importance of hookworms.. Am J Clin Nutr.

[19] Kabatereine NB, Brooker S, Koukounari A, Kazibwe F, Tukahebwa EM (2007). Impact of a national helminth control programme on infection and morbidity in Ugandan schoolchildren.. Bull World Health Organ.

[20] Koukounari A, Estambale BB, Njagi JK, Cundill B, Ajanga A (2008). Relationships between anaemia and parasitic infections in Kenyan schoolchildren: a Bayesian hierarchical modelling approach.. Int J Parasitol.

[21] Koukounari A, Fenwick A, Whawell S, Kabatereine NB, Kazibwe F (2006). Morbidity indicators of *Schistosoma mansoni*: relationship between infection and anemia in Ugandan schoolchildren before and after praziquantel and albendazole chemotherapy.. Am J Trop Med Hyg.

[22] Friedman JF, Kanzaria HK, McGarvey ST (2005). Human schistosomiasis and anemia: the relationship and potential mechanisms.. Trends Parasitol.

[23] Tolentino K, Friedman JF (2007). An update on anemia in less developed countries.. Am J Trop Med Hyg.

[24] Robertson LJ, Crompton DW, Sanjur D, Nesheim MC (1992). Haemoglobin concentrations and concomitant infections of hookworm and *Trichuris trichiura* in Panamanian primary schoolchildren.. Trans R Soc Trop Med Hyg.

[25] Stephenson LS, Latham MC, Ottesen EA (2000). Malnutrition and parasitic helminth infections.. Parasitology.

[26] Stephenson L (1993). The impact of schistosomiasis on human nutrition.. Parasitology.

[27] Stoltzfus RJ, Dreyfuss ML, Chwaya HM, Albonico M (1997). Hookworm control as a strategy to prevent iron deficiency.. Nutr Rev.

[28] Assis AM, Prado MS, Barreto ML, Reis MG, Conceicao Pinheiro SM (2004). Childhood stunting in Northeast Brazil: the role of *Schistosoma mansoni* infection and inadequate dietary intake.. Eur J Clin Nutr.

[29] Corbett EL, Butterworth AE, Fulford AJ, Ouma JH, Sturrock RF (1992). Nutritional status of children with schistosomiasis mansoni in two different areas of Machakos District, Kenya.. Trans R Soc Trop Med Hyg.

[30] Ferreira HS, Coutinho EM (1999). Should nutrition be considered as a supplementary measure in schistosomiasis control?. Ann Trop Med Parasitol.

[31] Friis H, Mwaniki D, Omondi B, Muniu E, Magnussen P (1997). Serum retinol concentrations and *Schistosoma mansoni*, intestinal helminths, and malarial parasitemia: a cross-sectional study in Kenyan preschool and primary school children.. Am J Clin Nutr.

[32] Brooker S, Miguel EA, Waswa P, Namunyu R, Moulin S (2001). The potential of rapid screening methods for *Schistosoma mansoni* in western Kenya.. Ann Trop Med Parasitol.

[33] Koukounari A, Sacko M, Keita AD, Gabrielli AF, Landoure A (2006). Assessment of ultrasound morbidity indicators of schistosomiasis in the context of large-scale programs illustrated with experiences from Malian children.. Am J Trop Med Hyg.

[34] Parker DR, Bargiota A, Cowan FJ, Corrall RJ (1997). Suspected hypoglycaemia in out patient practice: accuracy of dried blood spot analysis.. Clin Endocrinol (Oxf).

[35] Gorstein J, Sullivan K, Yip R, de Onis M, Trowbridge F (1994). Issues in the assessment of nutritional status using anthropometry.. Bull World Health Organ.

[36] Hanley JA, Negassa A, Edwardes MD, Forrester JE (2003). Statistical analysis of correlated data using generalized estimating equations: an orientation.. Am J Epidemiol.

[37] Beasley NM, Tomkins AM, Hall A, Kihamia CM, Lorri W (1999). The impact of population level deworming on the haemoglobin levels of schoolchildren in Tanga, Tanzania.. Trop Med Int Health.

[38] Ferreira MR, Souza W, Perez EP, Lapa T, Carvalho AB (1998). Intestinal helminthiasis and anaemia in youngsters from Matriz da Luz, district of Sao Lourenco da Mata, state of Pernambuco, Brazil.. Mem Inst Oswaldo Cruz.

[39] Gilgen DD, Mascie-Taylor CG, Rosetta LL (2001). Intestinal helminth infections, anaemia and labour productivity of female tea pluckers in Bangladesh.. Trop Med Int Health.

[40] Beaver PC (1975). Biology of soil-transmitted helminths: the massive infection.. Health Lab Sci.

[41] Lwambo NJ, Brooker S, Siza JE, Bundy DA, Guyatt H (2000). Age patterns in stunting and anaemia in African schoolchildren: a cross-sectional study in Tanzania.. Eur J Clin Nutr.

[42] Bhandari N, Bahl R, Taneja S (2001). Effect of micronutrient supplementation on linear growth of children.. Br J Nutr.

[43] Koukounari A, Gabrielli AF, Toure S, Bosque-Oliva E, Zhang Y (2007). *Schistosoma haematobium* infection and morbidity before and after large-scale administration of praziquantel in Burkina Faso.. J Infect Dis.

[44] Stoltzfus RJ, Albonico M, Tielsch JM, Chwaya HM, Savioli L (1997). Linear growth retardation in Zanzibari school children.. J Nutr.

[45] Simondon KB, Simondon F, Simon I, Diallo A, Benefice E (1998). Preschool stunting, age at menarche and adolescent height: a longitudinal study in rural Senegal.. Eur J Clin Nutr.

[46] Institut National de la Statistique du Rwanda (INSR) and ORC Macro (2006). Rwanda Demographic andHealth Survey 2005,.

[47] Webster JP, Koukounari A, Lamberton PHL, Stothard JR, Fenwick A (2009). Evaluation and application of potential schistosome-associated morbidity markers within large-scale mass chemotherapy programmes.. Parasitology: (Article in press).

[48] de Silva NR, Brooker S, Hotez PJ, Montresor A, Engels D (2003). Soil-transmitted helminth infections: updating the global picture.. Trends Parasitol.

[49] Fenwick A, Molyneux D, Nantulya V (2005). Achieving the Millennium Development Goals.. Lancet.

[50] Holland CV, Taren DL, Crompton DW, Nesheim MC, Sanjur D (1988). Intestinal helminthiases in relation to the socioeconomic environment of Panamanian children.. Soc Sci Med.

[51] Raso G, Vounatsou P, Gosoniu L, Tanner M, N'Goran EK (2006). Risk factors and spatial patterns of hookworm infection among schoolchildren in a rural area of western Cote d'Ivoire.. Int J Parasitol.

