# Supplementary material for: Polyparasite Helminth Infections and Their Association to Anaemia and Undernutrition in Northern Rwanda
Source: PLoS Negl Trop Dis. 2009 Sep 15;3(9):e517. doi: 10.1371/journal.pntd.0000517 (PMC2737105; doi:10.1371/journal.pntd.0000517)
Supplement: Alternative Language Abstract S1 — Translation of the abstract into French by MAD. (0.03 MB DOC) [file pntd.0000517.s001.doc]

**Résumé**

**Contexte :** La schistosomiase intestinale et les géo-helminthiases constituent des problèmes de santé publique majeurs pour une grande partie de l'Afrique sub-saharienne. Lors de cette étude, nous avons examiné l'implication fonctionnelle de telles infections poly-parasitaires sur le taux d'anémie et la sous-nutrition chez les rwandais.

**Méthodes :** Nous avons défini trois profils poly-parasitaires et un profil de référence, correspondant à l'absence d'infection ou une infection de faible intensité avec une seule des espèces de parasites étudiés ici. Afin d'identifier précisément les individus dont la probabilité d'être anémique et/ou sous-nourris est la plus grande, des modèles de régression logistique ont ensuite été appliqués aux données obtenues, avant traitement chimio-thérapeutique, sur 1 605 individus dans 6 écoles de 2 districts de la province Nord du Rwanda.

**Résultats :** Cette étude a permis de mettre en évidence une plus grande probabilité d'être anémique, indépendamment du profil d'infection poly-parasitaire, pour les individus atteints d'un retard de croissance clinique et les individus de sexe masculin que pour les individus à croissance normale et les individus de sexe féminin, respectivement. Par ailleurs, nous avons montré que la probabilité d'être cliniquement amaigri était 2 fois plus grande pour les enfants souffrant d'une coïnfection M+ avec au moins 2 espèces parasitaires que pour les enfants présentant le profil de référence. De plus, les données ont identifié que les individus de sexe masculin et les individus anémiques avaient une probabilité significativement plus élevée d'être atteints d'un retard de croissance clinique que les individus de sexe féminin et les individus non-anémiques, respectivement. Enfin, aucun des trois profils poly-parasitaires n'a exhibé une association significative avec le retard de croissance clinique.

**Conclusion :** Les données de cette étude suggèrent que les niveaux de poly-parasitisme et les intensités d'infection chez les individus rwandais examinés ici seraient plus bas que ceux présentés dans d'autres études épidémiologiques similaires récemment conduites dans d'autres régions de l'Afrique sub-saharienne. Nous n'avons pas trouvé de différence significative entre les trois profils d'infection poly-parasitaires étudiés quant à la probabilité d'être anémique, ou celle de souffrir d'un retard de croissance clinique. Cependant, la probabilité d'être cliniquement amaigri était plus élevée chez les enfants souffrant d'une coïnfection M+ avec au moins 2 espèces parasitaires que pour les enfants présentant le profil de référence. En dépit de la faible morbidité enregistrée chez les individus testés lors de cette étude, nous recommandons que des efforts de déparasitage pérennes des populations affectées soient maintenus afin de soutenir le développement économique du pays.
